# The Monitoring and Assessment of Aquatic Toxicology

**DOI:** 10.1155/2017/9179728

**Published:** 2017-01-03

**Authors:** Zongming Ren, Tae-Soo Chon, Chunlei Xia, Fengqing Li

**Affiliations:** ^1^Institute of Environment and Ecology, Shandong Normal University, Jinan 250014, China; ^2^Department of Biological Sciences, Pusan National University, Pusan, Republic of Korea; ^3^Yantai Coastal Zone Research Institute, Chinese Academy of Sciences, Beijing, China; ^4^Senckenberg Research Institute, Gelnhausen, Germany

Chemicals, including inorganic chemicals (nanoparticles, heavy metals, and other chemicals) and organic chemicals (pesticides, POPS, PBDEs, and other organic chemicals), are widely used in the world in recent 30 years to support the rapid development of industrialization and agriculture. The extensive use and discharge of these chemicals in these processes will produce wastewater containing a variety of contaminants, and the aquatic environment pollution will induce aquatic toxicology and may impair biological communities. However, due to the lack of target specificity, these chemicals can cause severe and persistent toxic effects on nontarget aquatic species, including bacteria, invertebrates, and vertebrates. Nowadays, knowledge and understanding of these conditions have led to the development of new monitoring, analysis, and assessment technologies based on biological and chemical methods. Therefore, it is very important to introduce these new methods to readers, which may include the characteristics of water pollutions, sampling techniques, chemical analysis methods, transport and transformation of chemicals, monitoring and assessment of aquatic toxicology, and the use of wastewater and other quality water.

The articles contained in this issue include both reviews and basic scientific studies focused on new monitoring technologies, mathematical analysis methods, and aquatic environment assessment of the aquatic toxicology.

Due to the widespread usage as flame retardants and the persistence of Polybrominated Diphenyl Ethers (PBDEs), they have become ubiquitous in the environment. In the “Environmental Characteristics of PBDEs in Marine System with Emphasis on Marine Organisms and Sediments” by Y. Zhang et al., authors provided an overview of the widespread of PBDEs in global scale and analyzed the concentrations of different PBDEs using different methods, for example, meta-analysis and principal component analysis.

Nanoparticles can affect the organisms in different environment. Relatively low concentrations of Silver nanoparticle (AgNP, 5 nm) will impact basic physiological functions. In the work by I. Saggese et al. entitled “Silver Nanoparticles Affect Functional Bioenergetic Traits in the Invasive Red Sea mussel* Brachidontes pharaonis,*” the authors showed that AgNP significantly affected the average respiration rate of* B. pharaonis*, and heart beat rates largely increased with no acclimation in* B. pharaonis* in both 20 *μ*g L^−1^ and 40 *μ*g L^−1^ AgNP. Eventually, a decreasing trend for absorption efficiency might indicate energetic constrains.

Differing from the land ecosystem and marine ecosystem in the structure and function, the mangrove wetland plays an extremely important role in the global ecological balance. As trace metals with low solubility in water are easily adsorbed and accumulated in sediments, coastal sediments are always regarded as the ultimate sinks for trace metals. In the paper “Environmental and Ecological Risk Assessment of Trace Metal Contamination in Mangrove Ecosystems: A Case from Zhangjiangkou Mangrove National Nature Reserve, China” by J. Wang et al., authors analyzed the concentrations, sources, and pollution assessment of trace metals (Cu, Cd, Pb, Cr, Zn, As, and Hg) in surface sediment from 29 sites and the biota specimen and made the assessment using Pollution load index and geoaccumulation index, which indicated unpolluted status in general except Pb, Cr, and As, in Zhangjiangkou Mangrove National Nature Reserve, China.

The evaluation and prediction of combined effect of chemical pollutants in the environment have become an interesting research topic in environmental chemistry. The paper “Quantitative Characterization of the Toxicities of Cd-Ni and Cd-Cr Binary Mixtures Using Combination Index Method” by L. Mo et al. investigated the toxic effect of binary mixture of Cd-Ni and Cd-Cr on* Chlorella pyrenoidosaosa* and* Selenastrum capricornutum*, and the concentration proportion of the mixture has greater influence on the type of interaction.

Phthalic acid esters (PAEs) are endocrine-disrupting pollutants and can show adverse effects on the environment and human health, inducing hepatic peroxisome proliferation, hormonal disorders, reproductive toxicity, and carcinogenicity. X. Zhang et al. described three freshwater unicellular organisms in the biodegradation of one kind of PAEs-dimethyl phthalate (DMP):* Synechocystis *sp.,* Synechococcus* sp., and* Cyanothece* sp., in the paper “Biodegradation of Dimethyl phthalate by Freshwater Unicellular Cyanobacteria.” It was reported that low concentration 20 mg L^−1^ DMP stimulated the growth of the three freshwater unicellular cyanobacteria, but higher concentration could inhibit the growth.

The Stepwise Behavior Response Model (SBRM) was proposed to address sequential behavior patterns during the course of response to the chemical. And the model included No effect, Stimulation, Acclimation, Adjustment (Readjustment), and Toxic effect, and similar behavior patterns were reported in macroinvertebrates. In the paper “Integrative Characterization of Toxic Response of Zebra fish* (Danio rerio)* to Deltamethrin based on AChE Activity and Behavior Strength” by Q. Ren et al., it was reported that the inhibition of acetylcholinesterase (AChE) activity by Deltamethrin is one of the factors that could affect the swimming behavior of aquatic organisms.

Worldwide industrialization and urbanization have enhanced the release of organic and inorganic pollutants in soil, water, and air.

Due to intensive human activities, coastal areas face serious ecosystem pressure. To achieve sustainable development, it is necessary to formulate measures to control pollution sources and protect the coastal environment. K. Chen et al. describe an optimization of environmental monitoring in the paper “Optimization of a coastal environmental monitoring network based on the Kriging method: a case study of Quanzhou Bay, China” using the Kriging method and a geographic information system. The results suggested that the monitoring network optimization was appropriate for environmental monitoring in Quanzhou Bay, and it could provide technical support for coastal management and pollutant reduction in similar areas.

